# A Polyketide Synthase Acyltransferase Domain Structure Suggests a Recognition Mechanism for Its Hydroxymalonyl-Acyl Carrier Protein Substrate

**DOI:** 10.1371/journal.pone.0110965

**Published:** 2014-10-23

**Authors:** Hyunjun Park, Brian M. Kevany, David H. Dyer, Michael G. Thomas, Katrina T. Forest

**Affiliations:** Department of Bacteriology, University of Wisconsin-Madison, Madison, Wisconsin, United States of America; Indian Institute of science, India

## Abstract

We have previously shown that the acyl transferase domain of ZmaA (ZmaA-AT) is involved in the biosynthesis of the aminopolyol polyketide/nonribosomal peptide hybrid molecule zwittermicin A from *cereus* UW85, and that it specifically recognizes the precursor hydroxymalonyl-acyl carrier protein (ACP) and transfers the hydroxymalonyl extender unit to a downstream second ACP via a transacylated AT domain intermediate. We now present the X-ray crystal structure of ZmaA-AT at a resolution of 1.7 Å. The structure shows a patch of solvent-exposed hydrophobic residues in the area where the AT is proposed to interact with the precursor ACP. We addressed the significance of the AT/ACP interaction in precursor specificity of the AT by testing whether malonyl- or methylmalonyl-ACP can be recognized by ZmaA-AT. We found that the ACP itself biases extender unit selection. Until now, structural information for ATs has been limited to ATs specific for the CoA-linked precursors malonyl-CoA and (2*S*)-methylmalonyl-CoA. This work contributes to polyketide synthase engineering efforts by expanding our knowledge of AT/substrate interactions with the structure of an AT domain that recognizes an ACP-linked substrate, the rare hydroxymalonate. Our structure suggests a model in which ACP interaction with a hydrophobic motif promotes secondary structure formation at the binding site, and opening of the adjacent substrate pocket lid to allow extender unit binding in the AT active site.

## Introduction

Fatty acids of various lengths and oxidation states are biosynthesized from malonyl-CoA and (2*S*)-methylmalonyl-CoA by fatty acid synthases (FASs). In contrast to FASs, the evolutionarily related polyketide synthases (PKSs), which catalyze the biosynthesis of the pharmaceutically important class of natural products called polyketides [1], are able to use a far greater repertoire of substrates [2]. The acquisition of this extended biosynthetic vocabulary by PKSs enables these enzymes to catalyze the formation of molecules with great structural and functional diversity. This diverse group includes molecules with antibacterial, antifungal, antitumor, and anticholesterol properties.

Given that PKSs descended from FASs, it is reasonable to assume that the substrates initially utilized by PKSs were limited to malonyl-CoA and (2*S*)-methylmalonyl-CoA. Coincidently, the PKSs that were first analyzed, and have therefore served as model systems for PKS research, only used these two molecules as substrates. However, the evolution of PKSs resulted in the inclusion of many more molecules as polyketide substrates, and in recent years our understanding of PKSs has also progressed past relatively simple systems to include PKSs that use this expanded substrate repertory to form highly specialized structures. Engineering previously characterized PKSs to incorporate non-cognate substrates containing unique functional groups, just as nature has done, is a significant goal in natural products research.

The effort to rationally reprogram PKSs to generate useful natural product analogs must begin with a solid foundation of basic PKS enzymology. PKSs are megasynthases that catalyze the decarboxylative Claisen condensation of various short carboxylic acid precursors, the first one referred to as the starter unit, and then extender units thereafter. Despite the vast structural diversity of polyketide molecules, PKSs (like FASs) comprise highly conserved discrete functional domains and linkers; each element plays a specific role such as recognition and incorporation, condensation, or modification of extender units [3]. The acyltransferase (AT) domain in PKSs is considered the gatekeeper domain because its function is to recognize a particular thioesterified extender unit with high specificity and to transacylate it onto a downstream acyl carrier protein (ACP) domain. This transacylation reaction proceeds via a ping-pong mechanism. The first half of the reaction consists of the AT receiving the extender unit from the carrier portion of the substrate, resulting in the esterification of the moiety on the side chain of the active site serine residue [1]. In the second half of the reaction, the extender unit is transferred from the active site serine residue of the AT onto the 4′-phosphopantetheinyl arm of the downstream ACP. This second step requires all ATs to make protein-protein interactions with their partner downstream ACP domains.

**Figure 1:**
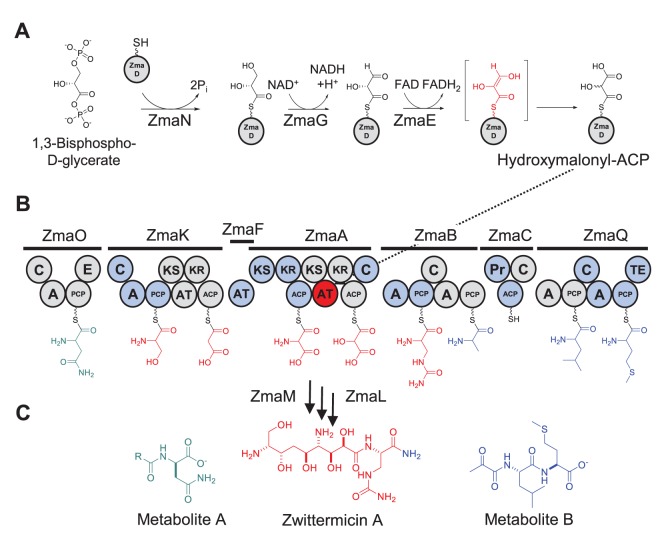
Biosynthesis of ZMA. (A) Biosynthetic pathway of hydroxymalonyl-ACP. The final FAD dependent oxidation step catalyzed by ZmaE may proceed through an endiol intermediate (red), resulting in the loss of stereospecificity at C2 of the final product, hydroxymalonyl-ACP. (B) ZMA PKS/NRPS. Nine extender units are utilized to form the precursor of metabolite A (green), zwittermicin A (red), and metabolite B (blue). Hydroxymalonyl-ACP is recognized by ZmaA (dotted line). Each circle represents a catalytic domain of the PKS/NRPS: C, condensation; A, adenylation; PCP, peptide carrier protein; E, epimerization; KS, ketosynthase; AT, acyltransferase; KR, ketoreductase; ACP, acyl carrier protein; Pr, protease; TE, thioesterase. (C) Natural prodrug activation. ZmaL is proposed to catalyze the cleavage of the ZMA precursor molecule from ZmaB-bound alanine, which is further condensed to leucine and methionine to form metabolite B (blue). ZmaM is proposed to catalyze the separation of metabolite A (green) from ZMA (red).

The majority of AT domains characterized so far are either malonyl-CoA or (2*S*)-methylmalonyl-CoA specific. More rarely, AT domains are specific for an ACP-tethered extender unit, such as methoxymalonyl-ACP, hydroxymalonyl-ACP, and aminomalonyl-ACP, the final two having been identified during our analysis of zwittermicin A (ZMA) biosynthesis (Figure 1A) [2]. For ATs that are specific for extender units carried by CoA, the substrate recognition step requires a protein-small molecule interaction between the AT and CoA, whereas for ATs specific for extender units with ACP carriers, this involves an additional protein-protein interaction.

ZMA is a polyketide/nonribosomal peptide hybrid antibiotic produced by *Bacillus cereus* strains UW85 and AH1134 that exhibits activity against a variety of Gram-negative and Gram-positive bacteria, as well as certain protists and plant pathogenic fungi [4]–[6]. Structural analysis of ZMA [7]–[9] revealed an aminopolyol structure with ethanolamine and glycolyl moieties that are rare in natural products, leading our group to focus on this biosynthetic aspect of ZMA. Based on our genetic and biochemical analyses (Figure 1B) [10]–[13] we proposed that ZMA biosynthesis involves the synthesis of an inactive larger molecule that is processed at both its amino and carboxy termini, releasing an amino-terminal acyl-D-aspartate (Figure 1C; metabolite A), the central ZMA molecule, and a carboxyl-terminal pyruvyl-L-leucyl-L-methionine (Figure 1C; metabolite B). Our proposed mechanism of ZMA activation by a D-amino acid peptidase, which cleaves the amino-terminal acyl-D-aspartate metabolite to release the active form of ZMA, was the first example of a natural prodrug biosynthetic scheme, also found to be involved in colibactin activation (Figure 1) [14]–[16]. Our analyses also revealed the existence of two rare PKS extender units aminomalonyl-ACP and hydroxymalonyl-ACP [12], and the AT domains that are specific for them, ZmaF and ZmaA-AT [13], respectively.

We have focused much of our analysis on the formation of hydroxymalonyl-ACP and aminomalonyl-ACP and the subsequent incorporation of the extender units by AT domains because the hydroxyl- and amino-groups originating from the C2 position of these extender units protrude away from the polyketide backbone, potentially serving critical functions or providing useful handles for downstream semi-synthetic modifications. For these reasons it is desirable to harness the ability to place these extender units in non-natural PKS settings. To do this, it is essential to understand how the respective AT domains recognize and incorporate these rare polyketide precursors.

Previous studies have identified four conserved regions that contribute to the molecular basis of AT substrate specificity [17]. In primary sequence order these are the RVDVVQ motif, the GHSXG motif centered on the active site serine residue, the YASH motif containing the histidine that is part of the catalytic dyad, and the last ∼30 residues of the AT domain (∼L378-S407). In addition to the four motifs that are implicated in extender unit recognition, RXR(X)_5_YASH has been implicated in the AT/substrate carrier recognition [18]–[22].

To further shed light on the substrate selection mechanism of these AT domains, we have solved the crystal structure of the hydroxymalonyl-ACP-specific ZmaA-AT domain. The structures of AT domains published to date include PKS AT domains involved in the biosynthesis of erythromycin A, pikromycin, dynemicin, and disorazole, as well as FAS AT domain homologs (malonyl-CoA:ACP transacylases) from *Escherichia coli* and *Streptomyces coelicolor* [18], [19], [21], [23]–[26]. Although these structures in the database reflect a considerable phylogenetic diversity, they are limited to recognizing malonyl-CoA or (2S)-methylmalonyl-CoA as their substrate. The structure presented here of a hydroxymalonyl-ACP-specific AT expands our understanding of AT domain recognition of ACP-linked extender units. The crystal structure of ZmaA-AT reveals an unusual solvent-exposed patch of hydrophobic residues in the proposed AT-ACP interaction surface. *In vitro* assays confirmed that this interaction plays a significant role in substrate recognition. The three-dimensional coordinates for ZmaA-AT allow us to compare the structure of an AT that is specific for an ACP-linked extender unit to the previously published structures of AT domains specific for CoA linked extender units that are involved in both polyketide and fatty acid biosynthesis. The crystal structure will be critical to the achievement of future PKS reprogramming efforts, where different substituents at the C2 position of the extender unit are desired for improved function or semi-synthetic amenability of the final PKS product.

## Materials and Methods

### Cloning of *zmaA* fragment *zmaA-AT*

The fragment of *zmaA* coding for the AT domain was cloned into *E. coli* expression vector pET-30a(+) (Novagen), using standard PCR-based cloning techniques, as described previously [13]. The following primers were used to introduce the gene fragment into the vector, resulting in the production of a protein containing an N-terminal histidine tag: 5′-GCACCAACCATGGAAGCAACATCAAATAGT-3′ and 5′-TATTTTCTCGAGAGACTACATTGGTAATGGGA-3′.

### Overproduction and purification of ZmaA-AT

pET-30a(+) containing *zmaA-AT* was introduced into *E. coli* Rosetta(DE3) (Novagen) and grown to an OD_600_ of 0.5 at 30**°**C, in lysogeny broth containing 50 µg/mL kanamycin and 15 µg/mL chloramphenicol. The temperature was reduced to 15**°**C and after 1 h overexpression was induced with IPTG at a final concentration of 60 µM. After 16 h, cells were harvested by centrifugation and the cell pellet was frozen at −20**°**C. The cell pellet was resuspended in buffer containing 20 mM Tris-HCl (pH 8.0), 300 mM NaCl and sonicated (Fisher 550 Sonic Dismembrator, power = 5, 15 min sonication with 1 s on, 1 s off). Sonicated cells were centrifuged for 30 min at 4°C to remove cell debris and insoluble protein (15,000 rpm, Beckman Model J221 centrifuge, JA-25.5 rotor, 4°C, for 30 min). ZmaA-AT was purified from the cell-free extract by nickel-affinity chromatography as previously described [27]. To enzymatically cleave the N-terminal histidine tag, the protein was concentrated to 15.5 mg/mL and dialyzed against buffer containing 20 mM Tris-HCl (pH8.0), 50 mM NaCl, and 2 mM CaCl_2_. Enterokinase (New England Biolabs) was added to dialyzed protein and incubated at room temperature (22**°**C) for 16 h. Enterokinase was removed by benzamidine-affinity chromatography (HiTrap Benzamidine FF, Amersham Biosciences). Fractions containing ZmaA-AT were collected and dialyzed against buffer containing 50 mM Tris-HCl (pH8.0) and 50 mM NaCl then concentrated to 6.7 mg/mL. ZmaA-AT was further purified by size-exclusion chromatography (Superdex 75, Amersham Biosciences). Fractions containing ZmaA-AT were pooled and concentrated to 7 mg/mL.

### Crystallization and Data Collection for ZmaA-AT

Initial crystallization conditions were obtained using vapour diffusion of protein (7 mg/ml) diluted with equal volume of mother liquor against the JCSG+ Suite screen (Qiagen). Crystal growth optimization resulted in final mother liquor of 100 mM BisTris pH 5.5, 200 mM MgCl_2_, 20% PEG 4000 and 800 mM sodium formate. Cryoprotection was achieved by soaking crystals in mother liquor plus 30% glycerol.

### Structure Determination

A 2.3 Å resolution native data set collected in house and processed with HKL-2000 [28] provided a highly significant molecular replacement solution using pdb code 2QO3 [23]. However, refinement was unsatisfactory. To overcome this issue ZmaA-AT was overproduced in the *E. coli* methionine auxotroph B834 (DE3) under conditions that led to incorporation of exogenously provided selenomethionine. The resulting selenomethionine-containing ZmaA-AT was purified to homogeneity. Crystals of this protein were obtained under similar conditions as the protein lacking selenomethionine. A 1.8 Å resolution data set was collected on the MAR 300 detector on beamline 21-ID-D at LS-CAT and processed with HKL-2000 [28]. The peak wavelength provided a strong anomalous signal, and Auto-rickshaw [29] was used to generate SAD phases. These were combined with a new partial molecular replacement model. Finally, a higher resolution native dataset was obtained, again on beamline 21-ID-D, and used for the ultimate refinement (Table 1). Refinement and fitting were carried out iteratively using REFMAC5 [30] and Coot [31] for final R_work_ and R_free_ values of 17.3 and 20.0%, respectively. Four amino acids at the N-terminus, one at the C-terminus, and three in an internal flexible loop were not observed. All structural images were generated using PyMOL [32].

**Table 1:** Data Collection and Refinement Statistics.

Data Collection Statistics		
Data Set	Native	SeMet Peak
Space Group	P2_1_2_1_2_1_	P2_1_2_1_2_1_
Unit Cell (Å)	a, b, c = 43.1, 73.2, 123.5	a, b, c = 47.0, 74.3, 125.5
Wavelength (Å)	0.97872	0.97934
Resolution* (Å)	25–1.65 (1.68–1.65)	30.0–1.84 (1.89–1.84)
Unique Reflections	54447 (2669)	37946 (2321)
Total Reflections	375883	514407
Completeness (%)	99.9 (100)	98.1 (85.3)
Redundancy	6.9 (6.0)	14.0 (8.3)
Average I/σ	27.7 (4.1)	46.9 (3.5)
R_sym_ a (%)	4.9 (36.3)	10.5 (41.7)
Wilson B value (Å^2^)	20.8	21.5
**Refinement Statistics**		
Resolution (Å)	25–1.70 (1.74–1.70)	
Reflections	46276 (3387)	
R**_cryst_** b (%)	17.3 (19.5)	
R**_free_** c (%)	20.0 (24.1)	
Average B value (Å^2^)	22.4	
Protein	21.6	
Ligand	42.5	
Water	28.2	
Est. Coord. Error (Å)	0.10	
Rmsd bonds (Å)	0.016	
Rmsd angles (°)	1.56	
Ramachandran plot		
Favored (%)	98.4	
Allowed (%)	99.8	
Outliers (%)	0.2	

*Highest resolution shells in parentheses.

a *R*_sym_(I) = ∑_hkl_∑_i_ |I_i_(*hkl*) – <*I*(*hkl*)>|/∑_hkl_∑_i_ I_i_(*hkl*) where *I*(*i*) is the intensity of the *i*th observation of the *hkl* reflection and <*I*(*hkl*)> is the mean intensity from multiple measurements of the *h*, *k*, *l* reflection.

b *R*_cryst_(*F*) = ∑*_hkl_*|*F*_obs_(*hkl*)–*F*_calc_(*hkl*)|/∑_hkl_ *F*_obs_(*hkl*), where *F*_obs_(*hkl*) and *F*_calc_(*hkl*) are the observed and calculated structure factor amplitudes for the *h*, *k*, *l* reflection.

c *R*_free_ is *R*_cryst_ calculated for a randomly selected test set of reflections (5%) not included in the refinement.

While preparing figures, we noticed the side chain of Leu192 of PDB entry 2G2Z [18] in an impossible orientation relative to the main chain and thus refitted the side chain to the publicly available data followed by a single round of real space refinement of residues 191–193 against these data using Coot.

### Radioactive Assays of ZmaA-AT with Malonyl-CoA, Methylmalonyl-CoA, Malonyl-ZmaD, and Methylmalonyl-ZmaD

To address the effect of the extender unit carrier on substrate recognition by the AT, ZmaA-AT was incubated with extender units with malonyl- or methylmalonyl- acyl groups on the carriers CoA or ZmaD (the ACP partner for ZmaA-AT) (Figure 1A). The reaction mixtures contained the following: 75mM Tris (pH 7.5), 10 mM MgCl_2_, 1 mM TCEP, 5 µM ZmaA-AT, and 1 µM Sfp (*Bacillus subtilis* phosphopantetheinyl transferase). 40 µM [^14^C-C2]malonyl-CoA (*malonyl-CoA) or 40 µM [^14^C-C2](2-*RS*)-methylmalonyl-CoA (*(2-*RS*)-methylmalonyl-CoA) was added to the reaction mixture either in the presence or absence of 5 µM ZmaD. Reaction mixtures were incubated for 1 h at 22**°**C and stopped with 50 µL of 2X cracking buffer [120 mM Tris-HCl (pH 6.8), 2% (v/v) β-mercaptoethanol, 1% (w/v) sodium dodecyl sulfate (SDS), 25% (v/v) glycerol, and 0.02% (w/v) bromophenol blue]. 30 µL was loaded onto a 15% polyacrylamide-SDS gel. The gel was stained with Coomassie Brilliant Blue, destained, dried, and exposed to a phosphorimaging screen and scanned with a Typhoon imager following a 4 day exposure. The scanned image was quantified using ImageJ [33] to determine the relative band intensities.

## Results

### Overall Structure of Zma-AT

**Figure 2:**
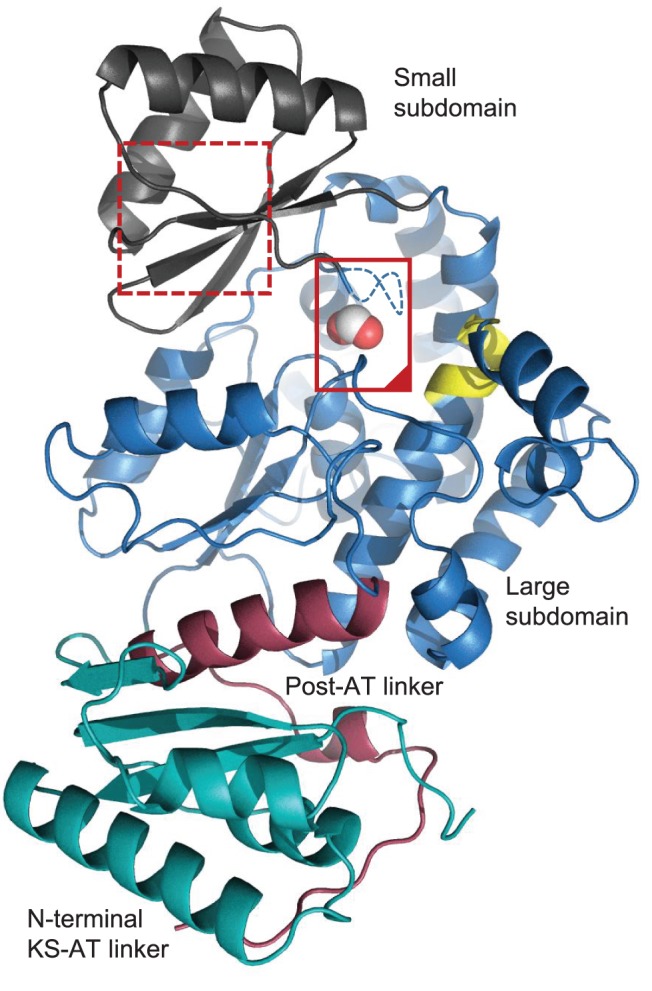
Overall structure of ZmaA-AT. The N-terminal KS-AT linker (green), α/β-hydrolase large subdomain (blue), small subdomain (gray), and post-AT linker (red) make up the complete asymmetric unit. The active site of ZmaA-AT (inside solid red box) is bounded on the left by the substrate pocket lid (containing the YASH motif, which in ZmaA-AT is GAAH) and on the top by the RVDVVQ motif (yellow) and is occupied by formate (spheres); cf. Figure 3. Residues E293-G294-A295 are not observed and are indicated with a dashed line. The proposed substrate ACP binding surface M286-E293 contains the methionine residues of the RXR motif (MCM in ZmaAT) (dotted red box) which correspond to the inchoate β-strand of the ferredoxin fold in the smaller subdomain of other ATs; cf. Figure 4.

The crystal structure of the ZmaA-AT domain, along with its N-terminal ketosynthase (KS)-AT linker, and 20 residue C-terminal post-AT linker, was refined against 1.7 Å resolution X-ray diffraction data (Table 1). The overall structure is similar to the analogous regions of the KS-AT domain pairs in modules 3 and 5 of the 6-deoxyerythronolide B (DEB) PKS from *Saccharopolyspora erythraea* (RMSD of 1.7 Å and 1.9 Å, respectively, for 308 and 301 C_α_ atom alignments against structures from PDB files 2QO3 and 2HG4) [23], [26]. Searches on 3-D BLAST [34] and the Dali Server [35] return the same two DEB PKS structures as the most significant structural matches. The ZmaA-AT domain (residues P93-S407) forms an α/β-hydrolase core into which a small subdomain is inserted (residues A226– I292) (Figure 2). As is the case with the two KS-AT didomain structures from the DEB PKS, the post-AT linker of ZmaA (residues D408-P443) wraps around the AT domain and makes extensive contacts with the highly ordered N-terminal KS-AT linker (residues T1-H92) (Figure 2).

We note that the overwhelming majority of AT domains that partner with CoA-bound extender units have a complete ferredoxin (βαββαβ) fold as the small subdomain [36]. In our formate-bound ZmaA-AT structure, those residues which would form the final β-strand do not make the required main chain hydrogen bonds to rigorously classify them as such (Figure 2). These amino acids, roughly spanning residues 286–291, immediately precede the residues that form a lid over the substrate pocket, both in primary sequence and 3D space (Figure 2).

### Motifs Implicated in Substrate Recognition

**Figure 3:**
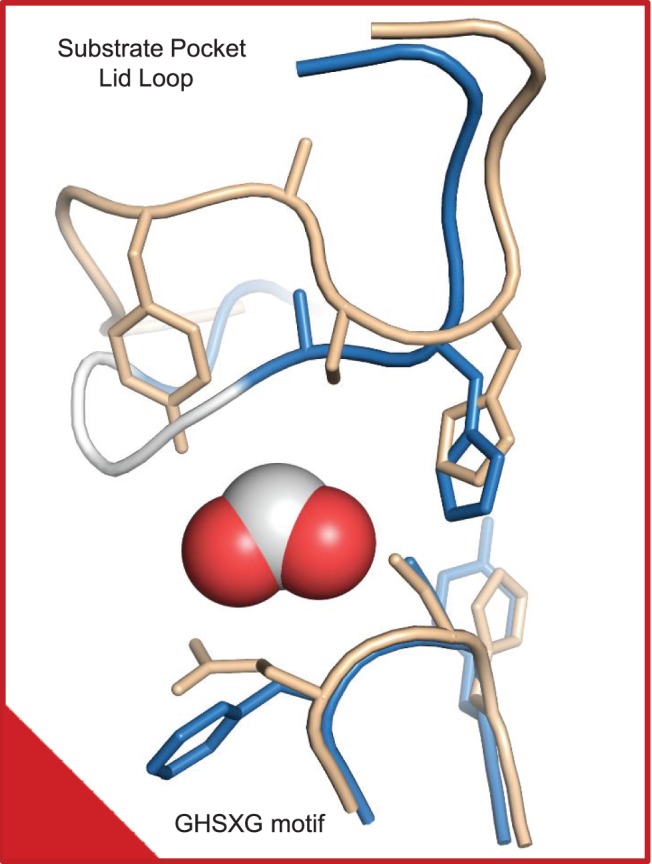
YASH and GHSXG motifs of ZmaA-AT compared to a methylmalonyl-CoA specific AT. The substrate binding-pocket amino acid residues (290–300 and 194) of ZmaA-AT (blue, with white span for disordered 293–295) are superimposed on those of AT from the DEB PKS module 3 (wheat). Bulky F193 is found next to the active site S192 in ZmaA-AT, instead of the glutamine residue found in methylmalonyl-CoA specific ATs. The catalytic H297 is positioned similarly to other ATs, despite its proposed steric hindrance to extender units with (2*R*) conformations. Despite high mobility for the substrate pocket lid YASH motif, we conclude based on the positions of well-ordered flanking residues that they must wander within the substrate binding pocket of ZmaA-AT, which holds co-crystallized formate (spheres). The red box, with its marked corner, can be compared to the same box in Figure 2 in order to orient the reader.

Structurally, the GHSXG (G190-YSF-G194 in ZmaA-AT), and the YASH (G294-AA-H297 in ZmaA-AT), motifs line the active site cleft formed between the two subdomains (Figure 3), while the RVDVVQ (R159-MEFS-Q164 in ZmaA-AT) motif forms a third wall of the active site and is positioned very close to the substrate pocket lid (Table 2, Figure 2). The structure does not inform how the C-terminal region of the AT domain indirectly influences substrate specificity and thus it will not be discussed in this report.

**Table 2:** The GHSXG and the YASH Motifs of Select Acyltransferases are Responsible for ACP *vs* CoA Discrimination.

AT Domain	Substrate	GHSXG Motif					YASH Motif			
ZmaA-AT	Hydroxymalonyl-ACP	G_190_	Y	S	F	G	G_294_	A	A	H
ZmaF	Aminomalonyl-ACP	G_90_	Y	S	L	G	G_198_	P	F	H
ZmaK-AT	Malonyl-CoA	G_1644_	H	S	I	G	H_1742_	A	F	H
FabD *(E. coli)*	Malonyl-CoA	G_90_	H	S	L	G	V_198_	P	S	H
FabD (*S. coelicolor*)	Malonyl-CoA	G_95_	H	S	V	G	G_198_	A	F	H
DEB PKS-AT3	Methylmalonyl-CoA	G_649_	H	S	Q	G	Y_751_	A	S	H
DEB PKS-AT5	Methylmalonyl-CoA	G_640_	H	S	Q	G	Y_742_	A	S	H

**Figure 4:**
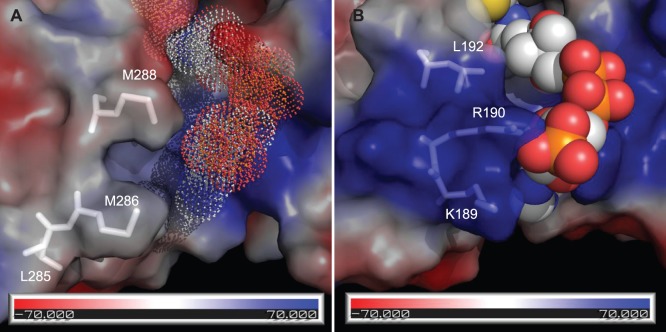
Proposed AT-Domain Interaction with ACP Substrate Carrier. Approximate protein contact potential calculated using PyMOL vacuum electrostatics function. The colors represent potentials ranging from −70 mV (red) to +70 mV (blue). (A) Proposed AT/ACP interface of ZmaA-AT. FabD was aligned to the structure of ZmaA-AT to show the relative position of CoA (dots, FabD not shown). (B) AT/CoA interface of *E. coli* FabD (PBD ID: 2G2Z, see Methods [18]). CoA is shown as spheres.

In ZmaA-AT, the region implicated in substrate carrier recognition is very similar to methoxymalonyl-ACP specific ATs and contains an MXW(X)_5_YASH motif (MXM(X)_5_GAAH in ZmaA-AT, Table 3) instead of the RXR(X)_5_YASH motif as ATs specific for CoA-tethered substrates [22]. ZmaA-AT structure features a hydrophobic patch in this region, instead of the positively charged surface as on the malonyl-CoA specific ATs (Figure 4).

**Table 3:** The RXR Motifs of Select Acyltransferases Control Extender Unit Specificity.

AT Domain	Substrate	(X)RXR				
ZmaA-AT	Hydroxymalonyl-ACP	(L)	M_286_	C	M	
ZmaF	Aminomalonyl-ACP	(G)	I_190_	A	I	
ZmaK-AT	Malonyl-CoA	(V)	K_1734_	T	T	
FabD *(E. coli)*	Malonyl-CoA	(K)	R_190_	A	L	
FabD (*S. coelicolor*)	Malonyl-CoA	(R)	K_190_	V	V	
DEB PKS-AT3	Methylmalonyl-CoA	(I)	R_743_	V	R	
DEB PKS-AT5	Methylmalonyl-CoA	(H)	K_734_	A	R	

### Alternative Substrate Recognition

We have previously established that ZmaA-AT recognizes hydroxymalonyl-ACP as its natural substrate, but it will also recognize aminomalonyl-ACP *in vitro*, when the AT is incubated with high concentrations of the latter [13]. The reduced activity of ZmaA-AT with aminomalonyl-ACP could be due to its specificity for the correct extender unit (aminomalonyl instead of hydroxymalonyl), the correct ACP (ZmaH instead of ZmaD), or a combination of both. Unfortunately the specificity of the enzymes that form aminomalonyl-ZmaH or hydroxymalonyl-ZmaD did not allow for the synthesis of hybrid precursors (e.g. aminomalonyl-ZmaD), thereby eliminating our ability to use these systems to test our hypothesis. Instead, we addressed the role of AT-ACP interaction by testing whether the AT domain can recognize the [^14^C-C2] labeled substrates *malonyl-CoA, *(2-*RS*)-methylmalonyl-CoA, *malonyl-ZmaD, and *(2-*RS*)-methylmalonyl-ZmaD *in vitro*, using Sfp (*Bacillus subtilis* phosphopantetheinyl transferase) to generate *malonyl-ZmaD and *(2-*RS*)-methylmalonyl-ZmaD from *malonyl-CoA, *(2-*RS*)-methylmalonyl-CoA, and apo-ZmaD.

**Figure 5:**
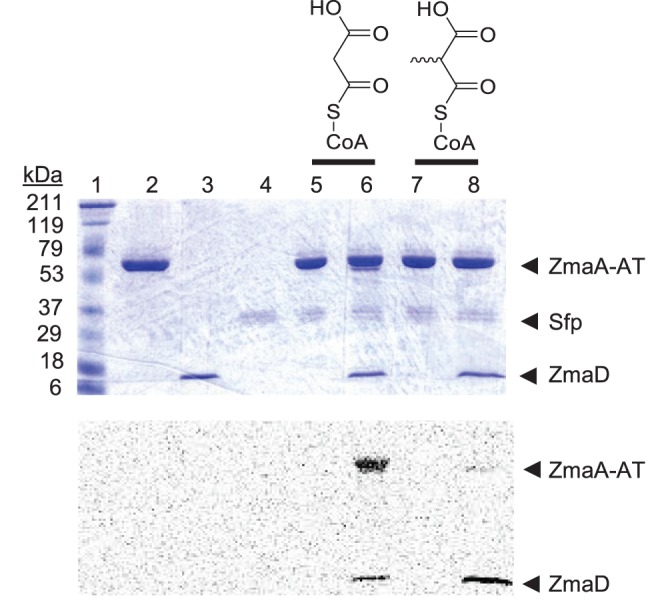
Transacylase assay of ZmaA-AT Distinguishes ACP from Acyl Unit Recognition. SDS-PAGE of reaction mixtures and corresponding phosphorimage. Lane 1: Molecular mass markers (Prestained Broad-range, Biorad). Lane 2: ZmaA-AT. Lane 3: ZmaD (ACP). Lane 4: Sfp (4′-phosphopantetheinyl transferase). Lane 5: ZmaA-AT, Sfp, and *Malonyl-CoA. Lane 6: ZmaA-AT, Sfp, ZmaD, and *Malonyl-CoA. Lane 7: ZmaA-AT, Sfp, and *(2-*RS*)-methylmalonyl-CoA. Lane 8: ZmaA-AT, Sfp, ZmaD, and *(2-*RS*)-methylmalonyl-CoA.

Neither *malonyl-CoA nor *(2-*RS*)-methylmalonyl-CoA was used by ZmaA-AT, whereas both *malonyl-ZmaD and *methylmalonyl-ZmaD were used to some extent, highlighting the importance of the AT-ACP interaction (Figure 5). Quantitative analysis revealed an average of ∼6 fold preference of *malonyl-ZmaD over *methylmalonyl-ZmaD, betraying an additional layer of substrate specificity at the AT-acyl unit interface. As a racemic mixture of *(2-*RS*)-methylmalonyl-CoA was used to generate methylmalonyl-ACP, it can be assumed that a racemic mixture of *(2-*RS*)-methylmalonyl-ZmaD was available to the AT. Since bacterial ATs associated with modular PKSs are known to be stereospecific [17], it is reasonable to estimate the difference in utilization of malonyl- and methylmalonyl-ACP by ZmaA-AT to be ∼3 fold.

## Discussion

### Overall Structure of ZmaA-AT

A notable difference between ZmaA-AT and previously reported AT domain structures is the positioning of the loop reconnecting the small subdomain to the large subdomain (residues I292-S298) (Figure 3). In all other AT structures published to date, this loop is positioned away from the substrate-binding pocket of the AT, whereas in the ZmaA-AT structure, it extends into the substrate-binding pocket. There aren’t any crystal packing interactions holding the lid in place. It is therefore reasonable to propose that binding of the substrate to the AT, especially the ACP portion of the substrate to the smaller subdomain, influences the positioning of this substrate pocket lid so that it moves out of the binding pocket to make room for the atoms of the extender unit. This hypothesis is supported by the high mobility of this region in ZmaA-AT. Indeed three residues (293–295) were poorly ordered and were omitted from the final model. The substrate pocket lid contains the YASH motif, which has been implicated in the extender unit specificity of AT domains (Table 2) [36].

**Figure 6:**
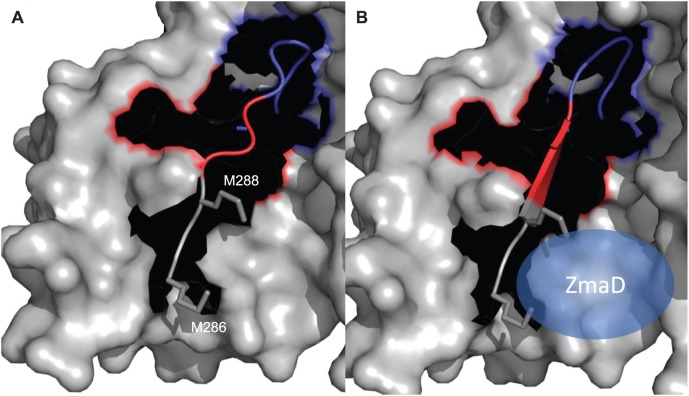
Proposed movement of the substrate pocket lid induced by ZmaD binding. (A) Based on the crystal structure of ZmaA-AT, the substrate pocket lid (blue) is shown in the closed position, restricting the entry of the extender unit, in the absence of substrate carrier protein. (B) Model structure of ZmaA-AT bound to substrate carrier protein, ZmaD (blue spheroid). The binding of the substrate carrier protein to the RXR motif (M286-C-M288 in ZmaA-AT; gray sticks) in the small subdomain of ZmaA-AT is proposed to cause the formation of the β-strand (red), resulting in the opening of the substrate pocket lid (blue).

We propose this motion is induced by the substrate carrier ACP binding to the RXR motif at the N-terminal end of this span of residues (M286-C-M288 in ZmaA-AT) (Figure 6). To date no substrate carrier ACP:AT co-crystal structures are available. Such a complex structure will be needed to validate this model of structural rearrangement upon ACP binding. The RXR motif is proposed to be involved in substrate carrier recognition and is discussed in detail below.

### AT Recognition of the Extender Unit

In ZmaA-AT, the highly conserved histidine in the GHSXG motif that includes the catalytic S192 is replaced with Y191 to form GYSFG (Figure 3, Table 2). However, the relative positioning of the phenol of Y191 to the catalytic S192 in ZmaA-AT matches that of the imidazole of the histidine and the catalytic serine in structures of FabD, DEB PKS-AT3 and DEB PKS-AT5, suggesting that they have a similar function [18], [23], [26]. The X following the catalytic serine in this motif is usually a bulky branched hydrophobic amino acid in ATs that recognize malonyl-CoA, whereas it is a glutamine in (2*S*)-methylmalonyl-CoA specific ATs (Table 2) [37]. It has been proposed that in (2*S*)-methylmalonyl-CoA specific ATs, the side chain of this glutamine may orient the incoming extender unit so that the α-methyl group is able to make a hydrophobic interaction with the tyrosine of the YASH motif [26]. In ZmaA-AT and in methoxymalonyl-ACP specific ATs FkbA-AT1 and FkbA-AT2 (involved in FK520 biosynthesis [38]), bulky hydrophobic amino acids such as phenylalanine (F193, Figure 3) or leucine are found instead of glutamine in the X of the GHSXG motif, respectively, similar to malonyl-CoA specific ATs (Table 2). The side chain of F193 in the ZmaA-AT structure is pointing away from the substrate-binding pocket (Figure 3), as is the side chain of L93 in the FabD structure. Without a change in side chain rotamer compared to these crystal coordinates, F193 would not affect the orientation of the incoming substrate.

The YASH motif, which is located about 100 residues beyond the GHSXG motif, contains the histidine residue of the catalytic dyad. The side chain of the residue has been proposed to play an important part in substrate specificity in addition to its catalytic role [39]. Along with the tyrosine residue (Y742 in DEB PKS-AT5), mentioned above in methylmalonyl-CoA specificity, the imidazole ring of the histidine residue in the YASH motif is proposed to sterically hinder the α-methyl group of a (2*R*)-methylmalonyl-CoA as it enters the active site, providing stereo selectivity for the (2*S*) stereoisomer [26]. This histidine residue is part of the catalytic dyad involved in the AT mechanism [40], a fact which leads to an interesting issue regarding the orientation of the extender unit α-substituent that can be utilized in polyketide metabolism.

**Figure 7:**
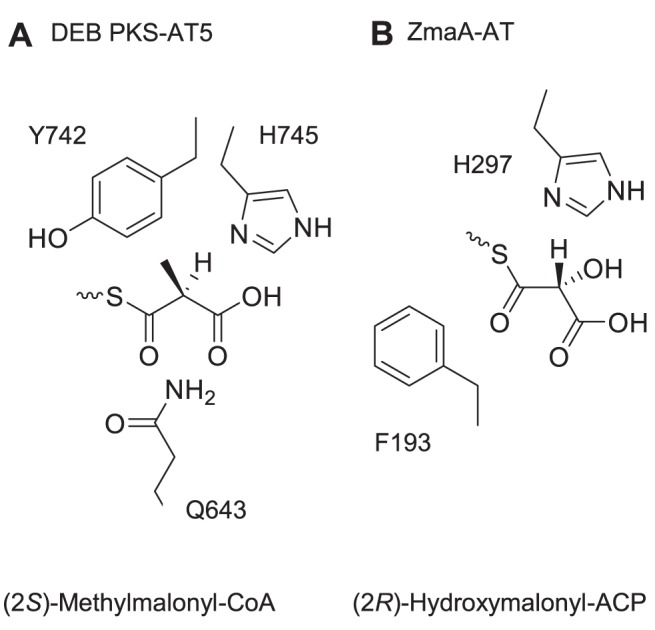
Possible difference in substrate entry angles between DEB PKS AT-5 and ZmaA-AT. (A) In DEB PKS-AT5, Q643 has been proposed to orient the incoming (2*S*)-methylmalonyl-CoA so that Y742 makes a hydrophobic interaction with the methyl-group and H745 sterically hinders the entry of (2*R*)-methylmalonyl-CoA [26]. (B) In ZmaA-AT, F193 is not positioned to orient the incoming substrate, which may allow hydroxymalonyl-ACP with (2*R*)-stereochemistry to enter the substrate pocket unhindered.

If ZmaA-AT were able to recognize (2*R*)-hydroxymalonyl-ACP, a hypothesis that is in keeping with the biosynthetic derivation from D-glycolytic intermediates, the stereochemistry of the extender unit would need to be reflected in the final (2*R*) product (Figure 1). In this case, the acyl moiety would be required to enter the substrate binding pocket at a significantly different angle than what is modeled for the (2*S*)-methylmalonyl-CoA entering the active site of DEB PKS-AT5 [26]. A different entrance pathway can be imagined because F193 in the GHSXG motif (GYSFG in ZmaA-AT) is positioned not to constrict the orientation of the substrate, and the α-hydroxyl group of the extender may not clash with the imidazole ring of H297 in the YASH motif (GAAH in ZmaA-AT) (Figure 7).

If the extender unit starts out in the (2*R*) conformation, after inversion from the condensation, there must be an additional epimerization event by the ketoreductase (KR) domain in ZmaA (ZmaA-KR2). ZmaA-KR2 does not contain an LDD motif (LGG in ZmaA-KR2) and its reduction reaction yields a hydroxyl group in the *S* conformation, suggesting it could be an A-type KR, by Caffrey classification [41]. However, because it also lacks an important tryptophan residue that is conserved in A type KRs, it belongs neither to the A1 nor the A2 KR type by Keatinge-Clay classification [42].

Alternatively, it is possible that ZmaA-AT recognizes the (2*S*)-isomer of hydroxymalonyl-ACP. The final step of hydroxymalonyl-ACP biosynthesis is an FAD-dependent oxidation of C3 by ZmaE [12]. Mechanistically, this step may proceed through an endiol intermediate (Figure 1A), which can then be re-protonated at C2 to form either the (2*S*) or (2*R*) stereoisomer of hydroxymalonyl-ACP. No epimerization would be required by the ZmaA-KR2 domain. In the ZMA molecule, the hydroxyl group at C8 is proposed to originate from the hydroxymalonyl-ACP extender unit incorporated by ZmaA-AT [11]. This hydroxyl group is in the same orientation as it would be on (2*R*)-hydroxymalonyl-ACP (Figure 1).

In the structure of FabD in complex with its substrate malonyl-CoA, the guanidine group of R117 is observed to stabilize the C3 carboxyl group of the acylated malonate through a salt bridge. The corresponding residue in our ZmaA-AT structure, R217, is positioned close to a molecule of formate, which co-crystallized with the protein and presumably mimics the coordinates of the C3 of hydroxymalonate. We note that the relative positioning of this R217 and the catalytic S192 of ZmaA-AT in solution would require less deviation from the crystal structure to accommodate the (2*S*) stereoisomer than the (2*R*) stereoisomer of hydroxymalonyl-ACP.

We conclude that there is presently not enough evidence to support the preference for one stereoisomer over the other in the incorporation of hydroxymalonyl-ACP by ZmaA-AT.

Finally, while the residues in the RVDVVQ motif would be too far away from the extender unit to contribute to substrate specificity directly, the structure suggests that amino acid substitutions in this motif may influence positioning of the YASH-motif in the substrate pocket lid, resulting in altered specificity [37].

### AT/Substrate Carrier Recognition

FabD is a malonyl-CoA specific AT in *E. coli,* involved in fatty acid synthesis. It must first interact with CoA to receive the malonyl group, then again with its partner downstream ACP to complete the transacylation reaction. Insight on the nature of the interaction between FabD and CoA was gained from the structure of FabD in complex with malonyl-CoA [18]. Later, the structure of a FabD homolog in *S. coelicolor* [19] was used for docking simulations using the structure of its partner downstream ACP [20]. These reports suggest that the arginine residues in FabD (R190 in *E. coli* and R189 in *S. coelicolor*) interact with and properly orient both the CoA and the downstream ACP. Similar results were obtained more recently, when crosslinking studies with the AT from the disorazole PKS and its partner ACP found that K179 on the AT is important for AT-ACP interaction [21]. K179 of the disorazole PKS-AT aligns with R189 of the *S. coelicolor* FabD. Interestingly, this region of the AT has also independently been implicated in substrate selectivity between methylmalonyl-CoA and methoxymalonyl-ACP [22]. In their work, Haydock *et al.* identified the sequence RXR(X)_5_YASH (the first Arg corresponds to R190 of *E. coli* FabD, Table 3) for methylmalonyl-CoA specific ATs, and MXW(X)_5_YASH for methoxymalonyl-ACP specific ATs within the concanamycin PKS. They noted that the methionine and tryptophan residues in MXW can be other hydrophobic residues in methoxymalonyl-ACP specific ATs, whereas these residues are usually replaced by positively charged ones in malonyl-CoA specific ATs. Using this sequence motif in a BLAST search, they were able to locate more ATs that are proposed to be methoxymalonyl-ACP specific.

Based on the similarity of ZmaA-AT to methoxymalony-ACP specific ATs in the region that is implicated in substrate carrier recognition, we propose that the signature motif MXW is indicative of not only methoxymalonyl-ACP specific ATs, but more generally, ATs that recognize ACP tethered extender units. This hypothesis is further supported by the fact that another AT domain involved in ZMA biosynthesis, ZmaF, recognizes an ACP tethered extender unit and contains hydrophobic residues in the MXW motif, while ZmaK-AT recognizes malonyl-CoA and has a positively charged residue in that motif (Figure 1 and Table 3). The exposed hydrophobic patch may facilitate an as yet uncharacterized binding of the AT with the extender unit ACP. This binding scheme would be distinct from the previously proposed transient electrostatic mode of interaction between the AT and the downstream ACP [20], [21]. Furthermore, as these hydrophobic residues lie in the region corresponding to the final β-strand of the ferredoxin fold in other ATs, we hypothesize that the binding of the substrate ACP to this region results in the formation of β-strand conformation in residues R284-T291 of the small subdomain, resulting in the displacement of the connected substrate pocket lid I292-S298 from inside the substrate binding pocket to accommodate the entry of the extender unit (Figure 6). Validation of this model awaits additional crystal structures of ACP specific ATs both alone and in complex with their ACP substrates.

### Alternative Substrate Recognition

The preference for malonyl-ACP over methylmalonyl-ACP as substrate by ZmaA-AT may be explained by the C2 methyl group of (2-*RS*)-methylmalonyl-CoA, which has a significantly larger radius than a hydroxyl group and may be sterically hindered by two tandem alanine residues (A295 and A296 in the GAAH). In addition, the methyl substituent restricts the bond angles of the backbone carbons of methylmalonate to be significantly different from those of hydroxymalonate. Therefore, when acylated on the active S192 of the AT, the C3 carboxyl group of the methylmalonyl extender unit would not be in the optimal position to form a salt bridge with R217, which is hypothesized to stabilize the C3 carboxyl group of the hydroxymalonyl extender unit. Unfortunately, efforts to substitute the Met residues in the RXR(X)_5_YASH of ZmaA-AT to positively charged residues resulted in insoluble protein, eliminating our ability to test whether such changes alter precursor recognition.

## Conclusion

PKS extender units that are biosynthesized on ACP carriers instead of CoA molecules include aminomalonyl and hydroxymalonyl moieties. These are of particular interest in combinatorial biosynthesis of polyketides, because their incorporation results in amino- and hydroxyl- functional groups, respectively, to be present at unique positions within the product, which can further be utilized in semi-synthetic derivatizations. Our bioinformatic and crystal structural analyses of ZmaA-AT as well as published structures of other AT domains have led to our hypotheses that (1) the presence of hydrophobic residues in the RXR motif of ATs indicates specificity for ACP tethered extender units and that (2) binding of the ACP to the hydrophobic patch promotes secondary structure formation of the β**-**strand that leads from the ACP binding site to the extender unit binding site, and opens the latter for substrate entry. *In-vitro* biochemical analysis of ZmaA-AT has shown that the AT/substrate ACP interaction plays a significant role in substrate specificity. Taken as a whole, this work establishes an important foundation for the engineering of ATs involving the utilization of ACP linked substrates.

### Accession Codes

The coordinates and structure factors have been deposited in the Protein Data Bank with accession code 4QBU.
